# Sedentary Behavior and Physical Activity Are Independent Predictors of Successful Aging in Middle-Aged and Older Adults

**DOI:** 10.1155/2012/190654

**Published:** 2012-09-11

**Authors:** Shilpa Dogra, Liza Stathokostas

**Affiliations:** ^1^School of Recreation Management and Kinesiology, Acadia University, Wolfville, NS, Canada B4P 2R6; ^2^Centre for Activity and Aging, University of Western Ontario, London, ON, Canada N6G 2M3

## Abstract

*Background*. Sedentary behavior is emerging as an important risk factor for poor health. Physical activity has proven to be important in determining overall successful aging (SA) among older adults; however, no data exists on the influence of sedentary behavior on SA. The purpose of this analysis was to determine whether there is an association between sedentary behavior and successful aging, independent of physical activity levels. *Methods*. 9,478 older (*M* = 4,245; *F* = 5,233) and 10,060 middle-aged (*M* = 4.621; *F* = 5,439) adults from the Healthy Aging cycle of the Canadian Community Health Survey were analyzed. Multivariate logistic regressions were conducted with SA and its three components as outcomes while physical activity and sedentary behavior were entered as main exposures. *Results*. Among older adults, compared to those who were sedentary (4 hours or more/day), those who were moderately (2–4 hours/day) and least sedentary (<2 hours/day) were 38% (OR: 1.38; CI: 1.12–1.69) and 43% (OR: 1.43; CI: 1.23–1.67) more likely to age successfully, respectively. Among middle-aged adults, those who were least sedentary were 43% (OR: 1.43; CI: 1.25–1.63) more likely to age successfully. *Conclusions*. These novel findings suggest that sedentary activities are significantly associated with lower odds of SA among middle-aged and older adults, potentially in a dose-dependent manner.

## 1. Introduction

 Recent research suggests that despite meeting the minimum physical activity recommendations, sitting for prolonged periods (i.e., sedentary behavior) can compromise the health of adults [1]. Literature has established that a physically inactive lifestyle and low levels of cardiorespiratory fitness lead to an increase in the risk of developing numerous chronic diseases as well as all-cause mortality [2]. Interestingly, sedentary behavior is emerging as a potentially important independent contributor to the relationship between lifestyle and health [3, 4]. 

Sedentary behavior is defined as any waking behavior characterized by an energy expenditure ≤1.5 METs and a sitting or reclining posture [5]. Accumulating evidence shows that, independent of physical activity levels, sedentary time is associated with an increased risk of cardiometabolic disease and all-cause mortality in children and adults [6–8]. Similar evidence is emerging for the older adult population. Katzmarzyk et al. [8] reported that all-cause death rates increased across daily sitting time categories in a dose-response manner in groups of adults under the age of 59 years and over 60 years. It has also been demonstrated that individuals greater than 60 years of age with metabolic syndrome spend a greater percentage of waking hours in sedentary time versus those with no metabolic disease [3]. Unfortunately, according to the 2007 Canadian Community Health Survey (CCHS), television viewing time increases steadily with age such that 36% of those aged from 55 to 64, 47% of adults from 65 to 74 years and 52% of adults 75 years and older spend 15 or more hours per week viewing television [6]. 

Beyond the influence on health, sedentary behavior may also influence overall successful aging (SA); a term used to represent the physical, psychological, and social success with which adults age. The relationship between physical activity and SA is already well established [9]. Unfortunately, there is limited data on the relationship between sedentary behavior and SA or the components of SA. There is also a dearth of data available on SA and physical activity in middle-aged adults. This information is critical as lifestyle behaviors have been shown to persist once they are developed [10–13]. For example, using a modified Rowe and Kahn [14] definition of SA, Sun et al. [15] showed that participants of the Nurse's Health Study surviving to age 70 who had higher levels of midlife physical activity had higher odds of “successful survival.” Similarly, in a 17-year longitudinal study, Britton et al. [11] found early-life exercise (mean age 44 years) to be a predictor of SA (free from major disease, good physical and mental function). No data exists on the relationship between overall SA and sedentary behavior in middle-aged adults. 

Recent reports indicate that 69% of waking hours of middle-aged and older adults are spent performing sedentary activities [16]. Given the strong relationship between physical activity and SA, an investigation between sedentary behavior and SA is warranted in this population. Clearly, if sedentary behavior is related to SA, middle-aged and older adult populations are at high risk of poor physical, psychological, and social health. The purpose of the present study therefore was to determine whether there is an association between sedentary behavior and SA, independent of physical activity, in a Canadian population of middle-aged and older adults.

## 2. Methods

### 2.1. Sample

The Healthy Aging cycle of the Canadian Community Health Survey (CCHS-HA) was used for the current analysis. The objective of the CCHS-HA is to provide information on SA, examine healthy aging from a multidisciplinary approach, examine the effects of lifestyle on age, and better understand the aging process in those aged 45 years and older. All data contained in this survey were self-reported and all participants provided informed consent prior to participation. The total sample size of the CCHS-HA is 30,865. For purposes of the current analysis the sample was restricted to those who had complete data for all variables of interest as outlined below (*n* = 19,538). Detailed information on data collection methods and data weighting can be found in the CCHS user guide [17].

### 2.2. Main Outcome

 SA variables were created for all three components of SA, that is, physical, psychological, and sociological. Young et al. [18] recently outlined the required variables to assess each of the components of SA based on Rowe and Kahn's model of SA [14]. Each SA component for the current analysis was based on this outline within the limitations of the database. 

#### 2.2.1. Physical Component of SA

The physical component of SA generally includes both the presence of chronic disease and functional impairments; however, Strawbridge et al. [19] showed that SA is not dependent on the presence or absence of disease and that many older adults were being misclassified based on this variable. Recent evidence using data in the CCHS indicates that self-perceived health is a better indicator of physical activity levels than the presence of chronic disease [20]. As such, the current analysis limited the definition of physical SA to those with functional impairment only. Participants were classified as having no mobility problems, having a problem but not requiring any aids, requiring mechanical support, or requiring help from others or cannot walk as per their responses to five separate questions in the CCHS-HA. Those in the first two groups were categorized as aging successfully and those in the latter two groups were categorized as aging poorly. 

#### 2.2.2. Psychological Component of SA

As per Young et al. [18] the psychological component should include data on cognitive function, emotional vitality, and depression. The CCHS-HA collected data on all three of these variables. Using two questions participants were classified into one of six categories for cognitive function: (1) able to remember most things, think clearly, and solve day-to-day problems, (2) able to remember most things but have a little difficulty when trying to think and solve day-to-day problems, (3) somewhat forgetful but able to think clearly and solve day-to-day problems, (4) somewhat forgetful and have a little difficulty when trying to think or solve day-to-day problems, (5) very forgetful and have great difficulty when trying to think or solve day-to-day problems, or (6) unable to remember anything at all and unable to think or solve day-to-day problems. Emotional vitality was based on a single question which classified participants as either (1) happy and interested in life, (2) somewhat happy, (3) somewhat unhappy, (4) very unhappy, or (5) so unhappy that life is not worthwhile. Finally, depression was assessed using a single question on the presence or absence of depression. Those in the first three categories of cognitive function and the first 2 categories of emotional vitality who did not have depression were classified as aging successfully in the psychological domain; all other were classified as aging poorly.

#### 2.2.3. Sociological Component of SA

 Engagement with life, social support, and spirituality are the main variables used to assess the sociological component of SA. Unfortunately there were no data pertaining to spirituality in the CCHS-HA. Two variables were used to classify participants as aging successfully and aging poorly, sense of belonging to the local community and the loneliness scale. Sense of belonging was a single question that classified participants as very strong, somewhat strong, somewhat weak, or very weak. The loneliness scale was based on three items: lack of companionship, feeling left out, and feeling isolated. Participants responded with either hardly ever, sometimes, or often. These scores were summed to create the loneliness scale. Those who had a strong sense of belonging and a loneliness scale score of ≤6 were classified as aging successfully, all other were classified as aging poorly.

#### 2.2.4. Overall SA

Those who were classified as aging successfully in the physical, psychological, and sociological domains were classified as aging successfully. All other were classified as aging poorly.

### 2.3. Main Exposures

#### 2.3.1. Physical Activity

 Unlike the other CCHS cycles, the CCHS-HA does not contain data on energy expenditure. The PA variable for the current analysis was therefore based on the number of hours the participant walked each week. Participants who spent 1 hour or more/day walking were classified as active, those who spent 30–60 minutes/day walking were classified as moderately active and those who spent <30 minutes/day walking were classified as inactive. 

#### 2.3.2. Sedentary Behavior

 The number of hours spent sitting per day were used to classify participants as sedentary (4 hours or more/day), moderately sedentary (2–4 hours/day), or least sedentary (<2 hours/day). 

### 2.4. Covariates

Middle-aged adults were those between the ages of 45 and 64 years, and older adults were those between the ages of 65 years and more. The CCHS-HA public access file does not contain age as a continuous variable for maintenance of confidentiality; therefore these data are presented in categories. Participants were classified as male or female based on self-reported biological sex. Marital status was categorized as either married/common-law, widowed/separated/divorced, or single/never married. Income was used as a proxy for socioeconomic status and was categorized as <20,000, $20–39,000, $40–59,000, $60–79,000, or >$80,000. These covariates were chosen based on previous literature [8, 19]. Marital status was additionally included as it is related to SA in older adults [20]. 

### 2.5. Statistical Analysis

Pearson chi-square analyses and standardized adjusted residuals that denote deviations from a normal distribution were [21] calculated to determine differences in all sample characteristics with the exception of age. Bivariate associations between each SA outcome and physical activity or sedentary behavior were conducted using logistic regression analysis for each sex and age group. Multivariate logistic regressions controlling for age, marital status, and income were conducted for each SA outcome with both physical activity and sedentary behavior entered in the model. These models were created for each sex and age group (middle-aged and older adults). All analyses were conducted using SPSS version 17.0 with statistical significance set at alpha < 0.05. In order to compensate for the deliberate over-sampling of particular groups, population weights supplied by Statistics Canada were applied to the entire dataset to ensure accurate population estimates. To estimate variance, the sample population weights were rescaled, standardized, and reapplied to the dataset. 

## 3. Results

Sample characteristics are described in Table 1. Overall there were significant differences between older and middle-aged adults for all covariates investigated. Interestingly there were no differences between overall SA between age groups, nor were there for the psychological component of SA for either of both sexes combined or for females.

Bivariate associations (Table 2) indicated a consistent trend for both physical activity and sedentary behavior such that those who were active and moderately active were significantly more likely to be aging successfully compared to those who were inactive. Similarly, those who were moderately sedentary and least sedentary were significantly more likely to be aging successfully compared to those who were sedentary. This trend was true for both sexes combined and for males and females separately. Additionally, in most cases there was a dose-response relationship such that those who were active or least sedentary had greater odds of SA than those who were moderately active or moderately sedentary, respectively.

Regressions adjusted for age, income, and marital status showed similar trends as bivariate regression (Table 3). Compared to inactive older adults, moderately active and active older adults were 41% (OR: 1.41; CI: 1.19–1.67) and 42% (OR: 1.42; CI: 1.20–1.69) more likely to be aging successfully overall, respectively. Similarly, compared to sedentary older adults, moderately sedentary and least sedentary older adults were 38% (OR: 1.38; CI: 1.12–1.69) and 43% (OR: 1.43; CI: 1.23–1.67) more likely to be aging successfully overall, respectively. This was similar to the results seen in middle-aged adults except that moderately sedentary adults were not more likely to be aging successfully overall compared to sedentary adults (OR: 1.08; CI: 0.96–1.21).

## 4. Discussion 

Using a sample of middle-aged and older adults from the CCHS-HA, we analyzed the relationship of SA with physical activity and sedentary behavior. Similar to previous research, we found that physical activity is strongly related to SA and each of its components. The novel findings of this study pertain to the association between sedentary behavior and SA. Our primary finding is that sedentary behavior is associated with SA such that those who spend less time in sedentary activities are more likely to age successfully, regardless of their physical activity levels. Our secondary finding is that the relationship between the physical component of SA with physical activity and sedentary behavior was stronger and occurred in a dose-response manner. Finally, for the psychological and sociological components of SA, it seems that sedentary behavior lasting <2 hours/day is required for SA. The present study is one of the first to highlight the adverse role of sedentary behavior in SA. These findings have implications for the development of sedentary guidelines for middle-aged and older adults.

Our finding that there is a strong association between physical activity and SA was as expected based on research pertaining to physical activity and SA. A direct association between SA and physical activity was noted by Baker et al. [9] using data from the CCHS (cycle 2.1, *n* = 12,042). They reported that only 11% of Canadians were aging successfully and that older adults who were physically active were 2.26 (estimate = 0.817, CI: 0.703–0.931) times more likely to age successfully compared to those who were physically inactive. In a follow-up study, Meisner et al. [21] showed that physical activity influences each component of SA, such that greater levels of physical inactivity were associated with an increased likelihood of reporting disease and disablement, low functional capacities, and being socially disengaged with life. While the results of these two studies imply that sedentary behavior would be associated with SA, no specific analyses to this effect were conducted. A study published by Ko et al. [22] showed that engaging in a greater number of activities (physical and nonphysical in nature) was significantly associated with several indicators of SA. Therefore this study shows that those who did not engage in activities (i.e., sedentary individuals) were less likely to age successfully. This is in direct line with the findings of our study. 

Physical activity is an established determinant of SA [23]. Moreover, the master athlete has been suggested as a model of SA given that this group of middle-aged and older adults is healthier and has a better quality of life than age-matched peers [24]. It is not surprising then that the strongest association in our study was found between sedentary behavior and the physical component of SA, that is, functional limitations. Several studies have shown that functional dependence is more likely to develop in older adults who are not physically active, or who were not physically active in middle age. Patel et al. [25] found that sedentary behavior in middle age had a significant impact on functional autonomy in older age using a population-based study. Similarly, Huang et al. [26] showed that middle-aged adults who were physically active and fit were less likely to have functional impairments in older age. These studies support our findings that middle-aged and older adults who were physically active and not sedentary were most likely to be aging successfully in the physical domain, that is, to maintain functional autonomy. 

In addition to a strong association between the physical component of SA and sedentary behavior, we also noted a dose-response relationship, that is, less time spent in sedentary activities was associated with higher odds of SA. In a recent review conducted on physical activity and functional limitations, a similar dose-response relationship was displayed such that those with higher levels of physical activity were less likely to develop functional limitations as compared to a sedentary group [27]. Spirduso and Cronin [28] conducted a review on the effect of the exercise dose response on SA using functional autonomy as a main outcome. The authors found that long-term physical activity was closely related to delaying disability and independent living in older adults. They also found that evidence for a dose response or a “threshold” between physical activity and physical functioning exists. It is difficult to assess a true dose response in the current analysis given the categorical nature of the variables in the data set. Whether there is indeed a threshold or a dose-response relationship should be determined in the future in order to develop optimal sedentary guidelines.

The possibility of a threshold for sedentary behavior was also observed for the psychological and sociological components of SA. Among older adults, the psychological component was not influenced by sedentary behavior whereas the sociological component was only influenced by sedentary behavior lasting less than two hours. Among the middle-aged adults, only those who were sedentary for less than two hours per day were more likely to age successfully, that is, those engaging in sedentary activities for 2–4 hours per day were not more likely to age successfully in these domains than those sedentary for 4 hours or more per day. In other words, less than two hours of sedentary activity per day may serve as a minimum duration (threshold) that must be achieved in order to age successfully in these two domains. This idea of a dose-dependent relationship or a threshold has been assessed in studies using physical inactivity and the psychological component of SA. Pietrelli et al. [29] found a dose-dependent effect of exercise on cognitive function and anxiety in an animal study using aerobic exercise in middle-aged and older rats. In the area of depression, a recent randomized control trial among adults aged 18–70 with depression found that the group who was assigned a higher dose of exercise had greater benefit than a group assigned a lower dose of exercise; however, both had clinically meaningful improvements with exercise participation [30]. There are also cross-sectional studies on the relationship between sedentary behavior and depression or hopelessness that support our findings. de Wit et al. [31] found that those who had depression and anxiety disorders were more likely to engage in sedentary activities such as television watching and computer use in a sample of adults aged 18–65. Similarly, among a group of middle-aged men, a negative association between engaging in physical activity and developing hopelessness was found such that those who engaged in higher volumes of physical activity were less likely to develop feelings of hopelessness [32]. With regards to the sociological component of SA, factors such as satisfaction with life [33], sense of belonging to community [34], and loneliness [35] are associated with physical inactivity, but again, little data exist on sedentary behavior. Future research should assess the dose-response relationship between sedentary activity and each component of SA. Furthermore, a consensus on the definition of SA should be reached.

### 4.1. Limitations

The current analysis has two limitations that are noteworthy. First, the CCHS-HA uses self-reported data; as such it is difficult to truly know how much time participants were spending in sedentary activities or being physically active. Therefore some participants may have been misclassified. Social desirability would dictate that physical activity was overreported and sedentary behavior was underreported. Given the broad categories used in the current analysis, it is less likely that such misclassification occurred. Second, the CCHS is a cross-sectional data set, so reverse causality cannot be ruled out. In other words, it cannot be said with certainty that sedentary behavior is causing poor outcomes as it is possible that poor outcomes are leading to sedentary lifestyles.

In conclusion, using a large database of middle-aged and older adults we found that similar to previous research, physical activity is strongly associated with SA. The novel finding of the current study is that sedentary behavior is significantly associated with lower odds of SA independent of physical activity levels, that is, sedentary behavior and physical activity may be independent risk factors for poor health among aging populations. We also found evidence for a dose-dependent relationship between sedentary behavior and each of the components of SA. Results of the present analysis are novel and have implications for the development of sedentary guidelines for middle-aged and older adults.

## Figures and Tables

**Table 1:** Characteristics of the sample by age group and sex.

				Older adults			Middle-aged adults	
			Both sexes	Males	Females	Both sexes	Males	Females
*n* (unweighted sample size)			9,478	4,245	5,233	10,060	4,621	5,439
	65–69 y	45–49 y	26.9	30.1	24.5	26.6	25.1	28.0
	70–74 y	50–54 y	19.8	21.2	18.6	30.8	34.3	27.5
Age	75–79 y	55–59 y	17.2	16.9	17.4	22.2	20.9	23.4
	80–84 y	60–64 y	12.8	9.9	15.0	20.4	19.6	21.0
	>80 y		23.3	21.8	24.4			

	>$20,000		24.1	13.7	32.2	8.3*	7.1*	9.5*
	$20,000–39,000		37.8	37.9	37.7	13.4*	11.3*	15.4*
Income	$40,000–59,000		18.3	21.3	15.9	17.2	15.5*	18.9*
	$60,000–79,000		8.9	11.1	7.2	18.6*	18.6*	18.7*
	>$80,000		10.9	16.0	7.0	42.4*	47.5*	37.5*

	Married/common law		48.7	67.0	34.4	74.9*	78.3*	71.8*
Marital status	Widowed		38.0	19.7	52.3	3.5*	1.5*	5.3*
	Separated/divorced		8.4	8.0	8.7	12.7*	10.6*	14.6*
	Single/never married		4.9	5.2	4.6	8.9*	9.6*	8.3*

	Active		33.3	36.4	30.9	39.3*	42.7*	36.2*
Physical activity levels	Moderately active		35.3	35.6	35.2	37.9*	35.7	40.1*
	Inactive		31.3	28.0	33.9	22.7*	21.6*	23.8*

	Least sedentary		14.9	15.7	14.3	25.2*	25.6*	24.9*
Sedentary behaviour	Moderately sedentary		33.8	34.4	33.3	34.0	33.2	34.7
	Sedentary		51.3	49.9	52.4	40.8*	41.2*	40.4*

Overall SA			56.8	58.0	55.8	56.9	58.1	55.6
Physical SA			87.6	90.4	85.5	98.4*	98.8*	98.1*
Psychological SA			85.3	84.9	85.7	86.6	89.2*	84.1
Sociological SA			70.5	70.8	70.2	63.2*	63.3*	63.2*

*significant differences between older and middle-aged adults within sex category.

*n*: sample size; SA: successful aging.

All data are weighted unless otherwise stated.

All data are a percent of the sample.

**Table 2:** Bivariate associations of successful aging with physical activity and sedentary behaviour.

				Older adults						Middle-aged adults			
Overall SA		Both sexes		Males		Females		Both sexes		Males		Females	
		OR	CI	OR	CI	OR	CI	OR	CI	OR	CI	OR	CI
	Inactive	1.00	Ref.	1.00	Ref.	1.00	Ref.	1.00	Ref.	1.00	Ref.	1.00	Ref.
Physical activity	Moderately active	1.60	1.36–1.89	1.34	1.08–1.57	1.39	1.16–1.67	1.37	1.20–1.56	1.34	1.08–1.57	1.39	1.16–1.67
	Active	1.69	1.43–1.99	1.30	1.08–1.57	1.58	1.31–1.90	1.44	1.27–1.65	1.30	1.08–1.57	1.58	1.31–1.90

	Sedentary	1.00	Ref.	1.00	Ref.	1.00	Ref.	1.00	Ref.	1.00	Ref.	1.00	Ref.
Sedentary behaviour	Moderately sedentary	1.58	1.36–1.84	0.91	0.77–1.07	1.31	1.11–1.54	1.09	0.97–1.23	0.91	0.77–1.07	1.31	1.11–1.54
	Least sedentary	1.60	1.31–1.95	1.22	1.02–1.46	1.63	1.36–1.94	1.41	1.24–1.61	1.22	1.02–1.46	1.63	1.36–1.94

				Older adults						Middle-aged adults			
Physical SA		Both sexes		Males		Females		Both sexes		Males		Females	
		OR	CI	OR	CI	OR	CI	OR	CI	OR	CI	OR	CI

	Inactive	1.00	Ref.	1.00	Ref.	1.00	Ref.	1.00	Ref.	1.00	Ref.	1.00	Ref.
Physical activity	Moderately active	2.64	1.66–4.17	3.22	1.51–6.84	2.34	1.30–4.19	2.64	1.66–4.17	3.22	1.51–6.84	2.34	1.30–4.19
	Active	4.03	2.4–6.78	5.81	2.44–13.86	3.07	1.60–5.90	4.03	2.4–6.78	5.81	2.44–13.86	3.07	1.60–5.90

	Sedentary	1.00	Ref.	1.00	Ref.	1.00	Ref.	1.00	Ref.	1.00	Ref.	1.00	Ref.
Sedentary behaviour	Moderately sedentary	1.91	1.20–3.05	2.83	1.19–6.73	1.61	0.92–2.82	1.91	1.20–3.05	2.83	1.19–6.73	1.61	0.92–2.82
	Least sedentary	2.61	1.45–4.68	1.90	0.83–4.34	3.44	1.48–7.97	2.61	1.45–4.68	1.90	0.83–4.34	3.44	1.48–7.97

				Older adults						Middle-aged adults			
Psychological SA		Both sexes		Males		Females		Both sexes		Males		Females	
		OR	CI	OR	CI	OR	CI	OR	CI	OR	CI	OR	CI

	Inactive	1.00	Ref.	1.00	Ref.	1.00	Ref.	1.00	Ref.	1.00	Ref.	1.00	Ref.
Physical activity	Moderately active	1.47	1.23–1.75	1.30	0.98–1.72	1.59	1.27–200	1.47	1.23–1.75	1.30	0.98–1.72	1.59	1.27–200
	Active	2.18	1.81–2.63	2.13	1.58–2.86	2.13	1.66–2.72	2.18	1.81–2.63	2.13	1.58–2.86	2.13	1.66–2.72

	Sedentary	1.00	Ref.	1.00	Ref.	1.00	Ref.	1.00	Ref.	1.00	Ref.	1.00	Ref.
Sedentary behaviour	Moderately sedentary	1.08	0.92–1.27	0.90	0.69–1.97	1.24	1.00–1.53	1.08	0.92–1.27	0.90	0.69–1.97	1.24	1.00–1.53
	Least sedentary	1.53	1.26–1.86	1.44	1.06–1.97	1.59	1.24–2.05	1.53	1.26–1.86	1.44	1.06–1.97	1.59	1.24–2.05

				Older adults						Middle-aged adults			
Sociological SA		Both sexes		Males		Females		Both sexes		Males		Females	
		OR	CI	OR	CI	OR	CI	OR	CI	OR	CI	OR	CI

	Inactive	1.00	Ref.	1.00	Ref.	1.00	Ref.	1.00	Ref.	1.00	Ref.	1.00	Ref.
Physical activity	Moderately active	1.32	1.15–1.51	1.19	0.98–1.46	1.44	1.20–1.73	1.32	1.15–1.51	1.19	0.98–1.46	1.44	1.20–1.73
	Active	1.32	1.15–1.51	1.14	0.94–1.38	1.51	1.25–1.83	1.32	1.15–1.51	1.14	0.94–1.38	1.51	1.25–1.83

	Sedentary	1.00	Ref.	1.00	Ref.	1.00	Ref.	1.00	Ref.	1.00	Ref.	1.00	Ref.
Sedentary behaviour	Moderately sedentary	1.03	0.92–1.16	0.89	0.75–1.05	1.19	1.01–1.41	1.03	0.92–1.16	0.89	0.75–1.05	1.19	1.01–1.41
	Least sedentary	1.37	1.20–1.56	1.25	1.03–1.51	1.50	1.24–1.80	1.37	1.20–1.56	1.25	1.03–1.51	1.50	1.24–1.80

SA: successful aging; OR: odds ratio; CI: confidence interval.

**Table 3:** Adjusted regressions of successful aging with physical activity and sedentary behaviour.

		Older adults						Middle-aged adults					
Overall SA		Both sexes		Males		Females		Both sexes		Males		Females	
		OR	CI	OR	CI	OR	CI	OR	CI	OR	CI	OR	CI
	Inactive	1.00	Ref.	1.00	Ref.	1.00	Ref.	1.00	Ref.	1.00	Ref.	1.00	Ref.
Physical activity	Moderately active	1.41*	1.19–1.67	1.62*	1.24–2.10	1.27*	1.02–1.58	1.35*	1.18–1.54	1.36*	1.11–1.66	1.33*	1.10–1.59
	Active	1.42*	1.20–1.69	1.57*	1.21–2.05	1.33*	1.06–1.68	1.45*	1.27–1.66	1.35*	1.12–1.64	1.54*	1.28–1.86

	Sedentary	1.00	Ref.	1.00	Ref.	1.00	Ref.	1.00	Ref.	1.00	Ref.	1.00	Ref.
Sedentary behaviour	Moderately sedentary	1.38*	1.12–1.69	1.36*	1.08–1.72	1.49*	1.22–1.83	1.08	0.96–1.21	0.90	0.76–1.07	1.27*	1.08–1.50
	Least sedentary	1.43*	1.23–1.67	1.20	0.88–1.62	1.56*	1.18–2.07	1.43*	1.25–1.63	1.23*	1.01–1.48	1.65*	1.37–1.98

		Older adults						Middle-aged adults					
Physical SA		Both sexes		Males		Females		Both sexes		Males		Females	
		OR	CI	OR	CI	OR	CI	OR	CI	OR	CI	OR	CI

	Inactive	1.00	Ref.	1.00	Ref.	1.00	Ref.	1.00	Ref.	1.00	Ref.	1.00	Ref.
Physical activity	Moderately active	1.62*	1.26–1.08	1.65*	1.09–2.51	1.61*	1.18–2.21	2.46*	1.53–3.94	2.90*	1.34–6.30	2.24*	1.35–7.55
	Active	1.80*	1.37–2.37	1.96*	1.25–3.07	1.75*	1.24–2.48	3.87*	2.28–6.58	5.63*	2.33–13.63	3.18*	1.62–6.22

	Sedentary	1.00	Ref.	1.00	Ref.	1.00	Ref.	1.00	Ref.	1.00	Ref.	1.00	Ref.
Sedentary behaviour	Moderately sedentary	1.75*	1.37–2.24	1.85*	1.22–2.79	1.69*	1.24–2.31	1.68*	1.04–2.72	2.28	0.93–5.56	1.50	0.84–2.66
	Least sedentary	2.44*	1.64–3.65	2.48*	1.28–4.79	2.45*	1.47–4.07	2.15*	1.18–3.92	1.27*	0.54–3.02	3.20*	1.62–6.22

		Older adults						Middle-aged adults					
Psychological SA		Both sexes		Males		Females		Both sexes		Males		Females	
		OR	CI	OR	CI	OR	CI	OR	CI	OR	CI	OR	CI

	Inactive	1.00	Ref.	1.00	Ref.	1.00	Ref.	1.00	Ref.	1.00	Ref.	1.00	Ref.
Physical activity	Moderately active	1.53*	1.22–1.91	1.96*	1.40–2.76	1.26	0.93–1.70	1.40*	1.17–1.68	1.27	0.95–1.71	1.46*	1.15–1.85
	Active	1.74*	1.37–2.21	2.47*	1.73–3.54	1.33	0.96–1.83	2.16*	1.78–2.63	2.29*	1.69–3.12	2.03*	1.58–2.62

	Sedentary	1.00	Ref.	1.00	Ref.	1.00	Ref.	1.00	Ref.	1.00	Ref.	1.00	Ref.
Sedentary behaviour	Moderately sedentary	1.08	0.82–1.44	1.03	0.75–1.42	1.40*	1.05–1.88	1.02	0.86–1.21	0.82	0.62–1.07	1.15	0.92–1.44
	Least sedentary	1.21	0.98–1.50	0.89	0.59–1.35	1.28	0.86–1.91	1.51*	1.23–1.85	1.30*	0.94–1.80	1.60*	1.23–2.08

		Older adults						Middle-aged adults					
Sociological SA		Both sexes		Males		Females		Both sexes		Males		Females	
		OR	CI	OR	CI	OR	CI	OR	CI	OR	CI	OR	CI

	Inactive	1.00	Ref.	1.00	Ref.	1.00	Ref.	1.00	Ref.	1.00	Ref.	1.00	Ref.
Physical activity	Moderately active	1.19*	1.00–1.43	1.30	0.98–1.72	1.11	0.88–1.40	1.30*	1.14–1.49	1.21	0.99–1.48	1.39*	1.15–1.67
	Active	1.21*	1.01–1.46	1.23	0.93–1.63	1.21	0.94–1.55	1.33*	1.16–1.52	1.17	0.96–1.43	1.49*	1.23–1.80

	Sedentary	1.00	Ref.	1.00	Ref.	1.00	Ref.	1.00	Ref.	1.00	Ref.	1.00	Ref.
Sedentary behaviour	Moderately sedentary	1.32*	1.12–1.56	1.17	0.85–1.62	1.30*	1.04–1.61	1.03	0.91–1.16	0.90	0.76–1.08	1.17	0.99–1.38
	Least sedentary	1.25*	1.00–1.55	1.35*	1.05–1.74	1.32	0.98–1.78	1.39*	1.22–1.59	1.28*	1.05–1.55	1.52*	1.26–1.84

SA: successful aging; OR: odds ratio; CI: confidence interval.

Adjusted for age, marital status, and income.
